# Supplementary material for: WEDAP: A Python Package for Streamlined Plotting of Molecular Simulation Data
Source: J Chem Inf Model. 2024 Jul 16;64(15):5749–55. doi: 10.1021/acs.jcim.4c00867 (PMC11323263; doi:10.1021/acs.jcim.4c00867)
Supplement: Supplementary file 1 — ci4c00867_si_001.pdf [file ci4c00867_si_001.pdf]

Supporting Information for: WEDAP: A Python Package for Streamlined Plotting of Molecular Simulation Data

Darian T. Yang[†,‡,¶] and Lillian T. Chong[*,¶,§]

†*Molecular Biophysics and Structural Biology Graduate Program, University of Pittsburgh and Carnegie Mellon University, Pittsburgh, Pennsylvania 15260*

‡*Department of Structural Biology, University of Pittsburgh School of Medicine, Pittsburgh, Pennsylvania 15260*

¶*Department of Chemistry, University of Pittsburgh, Pittsburgh, Pennsylvania 15260*

§*Corresponding author*

E-mail: ltchong@pitt.edu

Supporting Figures

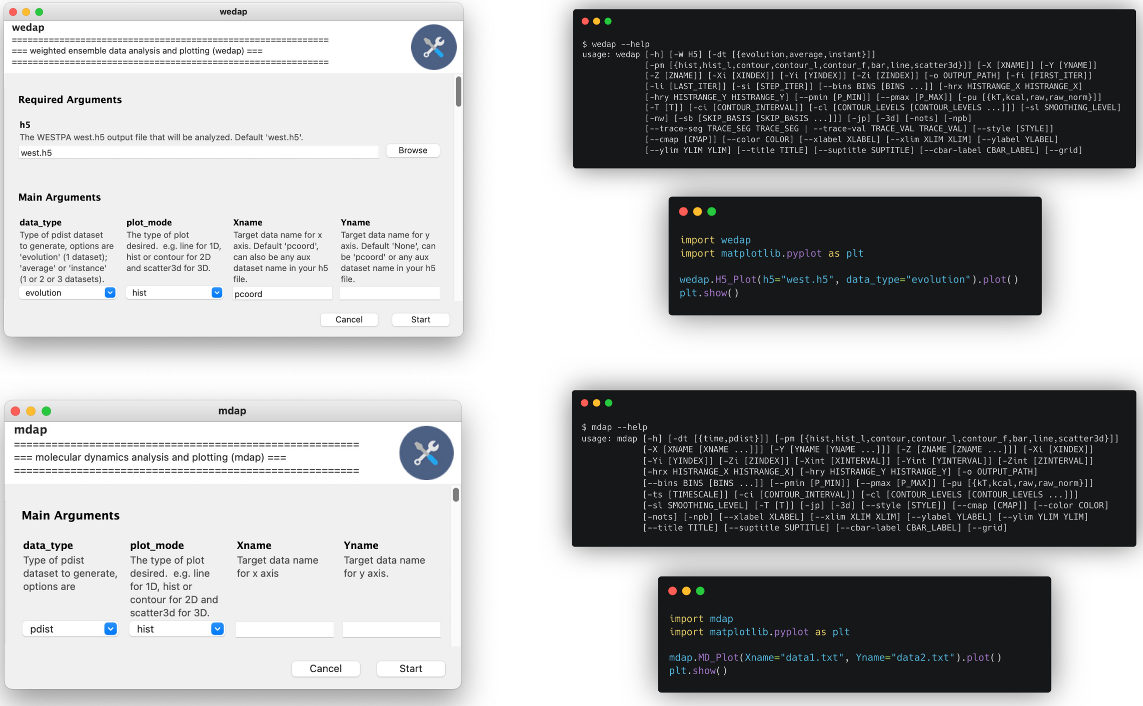

Figure S1: Within the WEDAP toolkit, `wedap`, `mdap`, and `wekap` are all available to use through a graphical user interface, the command line, or using the Python API. An example of each is shown for `wedap` (top) and `mdap` (bottom). The respective interfaces for `wekap` are not shown for simplicity.

```
/
    ibstates/
        index
        naming
            bstate_index
            bstate_pcoord
            istate_index
            istate_pcoord
    tstates/
        index
    bin_topologies/
        index
        pickles
    iterations/
        iter_XXXXXXXX/
            auxdata/
            bin_target_counts
            ibstates/
                bstate_index
                bstate_pcoord
                istate_index
                istate_pcoord
            trajectories
            pcoord
            seg_index
            wtgraph
        ...
    summary
```

Figure S2: The file organization structure of the output H5 file from WESTPA.
